# The molecular mechanism of LncRNA34a-mediated regulation of bone metastasis in hepatocellular carcinoma

**DOI:** 10.1186/s12943-019-1044-9

**Published:** 2019-07-26

**Authors:** Li Zhang, Hao Niu, Jie Ma, Bao-Ying Yuan, Yu-Han Chen, Yuan Zhuang, Gen-Wen Chen, Zhao-Chong Zeng, Zuo-Lin Xiang

**Affiliations:** 1 Department of Radiation Oncology, Zhongshan Hospital, Fudan University, 180 Feng Lin Road, Shanghai, 200032 China; 2 Department of Radiation Oncology, Shanghai East Hospital, Tongji University School of Medicine, 150 Jimo Road, Shanghai, 200120 China

**Keywords:** Hepatocellular carcinoma, Bone metastasis, Lnc34a, miR-34a, Smad4

## Abstract

**Background:**

Bone metastasis (BM) has long been recognized as a major threat to the quality of life of hepatocellular cancer (HCC) patients. While LncRNA34a (Lnc34a) has been shown to regulate colon cancer stem cell asymmetric division, its effect on HCC BM remains unknown.

**Methods:**

In situ hybridization and quantitative real-time polymerase chain reaction (qRT-PCR) were used to detect the expression of Lnc34a in HCC tissues and cell lines. Ventricle injection model was constructed to explore the effect of Lnc34a on BM in vivo. The methylation of miR-34a promoter and histones deacetylation were examined by using bisulfate-sequencing PCR and chromatin immunoprecipitation assays. RNA pull down and RNA immunoprecipitation were performed to investigated the interaction between Lnc34a and epigenetic regulators. Dual-luciferase reporter assay was conducted to find miR-34a target. The involvement of TGF-β pathway in the BM from HCC was determined by qRT-PCR, western, and elisa assays.

**Results:**

We found that Lnc34a was significantly overexpressed in HCC tissues and associated with BM. Both in vitro and in vivo experiments indicate that the restoration or knockdown of Lnc34a expression in HCC cells had a marked effect on cellular migration, invasion, and metastasis. Mechanistic analyses suggested that Lnc34a epigenetically suppresses miR-34a expression through recruiting DNMT3a via PHB2 to methylate miR-34a promoter and HDAC1 to promote histones deacetylation. On the other hand, miR-34a targets Smad4 via the TGF-β pathway, followed by altering the transcription of the downstream genes (i.e., CTGF and IL-11) that are associated with BM.

**Conclusions:**

Our study is the first to document the pro-bone metastatic role of Lnc34a in BM of HCC and reveal a novel mechanism for the activation of the TGF-β signaling pathway in HCC BM, providing evidence of a potential therapeutic strategy in HCC BM.

**Electronic supplementary material:**

The online version of this article (10.1186/s12943-019-1044-9) contains supplementary material, which is available to authorized users.

## Background

Hepatocellular cancer (HCC) is the most common primary liver cancer, accounting for 80–90% of cases, particularly in sub-Saharan Africa and Eastern Asia [1, 2]. With an estimated 782,000 newly diagnosed cases and 746,000 deaths worldwide in 2012, HCC is ranked the sixth most frequent cancer and the third leading cause of cancer-related death [2]. Recently, significant advances in the early diagnosis and novel treatment for HCC have been achieved, prolonging the survival of patients with HCC [3]. Metastases, the other hand, accompanied such long-term survival and occured during tumor progression [4]. Moreover, bone metastasis (BM) has been reported in approximately 38.5% of HCC patients with extrahepatic metastases [5, 6], and accounted for 11.7% of the HCC patients that underwent curative resections [7]. Additionally, HCC patients with BM suffered from severe pain, pathological fractures, and other nerve compression syndromes [8], with a median survival period of 7.4 months [9]. Thus, a better understanding of the pathogenetic mechanisms underlying the spread of BMs in HCC can facilitate the improvement of patient prognosis.

Long noncoding RNAs (lncRNAs) are functionally defined as transcripts greater than 200 nucleotides in length with limited or no protein coding potential [10]. Recently, several studies have shown that aberrant lncRNA expression is related to a variety of biological processes, including cellular proliferation [11], angiogenesis [12], and metastasis [13]. However, the role of lncRNAs in BM is poorly understood [14, 15]. The majority of lncRNAs are found in the cell nucleus [16] where they regulate gene expression via epigenetic modifications, on both a transcriptional and post-transcriptional level [17]. Lnc34a is a newly characterized lncRNA consisting of 693 bp that has no protein coding potential and epigenetically silences miR-34a expression, thereby initiating the asymmetric division of colon cancer stem cells [18]. However, the role of Lnc34a in BM in the context of HCC remains unknown.

In the present study, we found that Lnc34a contributes to BM in HCC via epigenetically suppressing miR-34a expression. We further identified miR-34a to inhibit BM through the regulation of TGF-β/Smad signaling. These findings may provide a novel diagnostic target and therapeutic strategy for the clinical prevention of BM from HCC.

## Methods

### Statistical analyses

The statistical analysis was performed using SPSS version 25.0 for Windows (SPSS, Chicago, USA). All data were expressed as the mean ± SD and repeated from at least three independent experiments. The difference between groups was analyzed using a Student’s *t*-test when comparing only two groups or a one-way analysis of variance when comparing more than two groups. Pearson χ^2^ test or Fisher exact test was used to compare qualitative variables. The potential association between the level of expression of the candidate molecules and the incidence of BM was analyzed with a log-rank test and Cox regression model. *P* < 0.05 was considered statistically significant (two-sided).

### Supporting materials and methods

For details regarding the patients, cell transduction, plasmids, luciferase reporter assays, TMA, immunohistochemistry (IHC), in situ hybridization (ISH), RNA isolation and qRT-PCR, RNA fluorescence in Situ Hybridization (FISH), cell proliferation, scratch assay, migration and invasion assay, western blot analysis, enzyme-linked immunosorbent assay (ELISA), dual luciferase reporter assay, bisulfate-sequencing PCR, RNA immunoprecipitation (RIP) and chromatin immunoprecipitation (ChIP) assays, intracardiac injections, bioluminescence assay, SPECT bone imaging, micro-CT, pathology analysis, tartrate-resistant acid phosphatase (TRAP), and other related procedures, refer to the Supporting Materials (Additional file 1: Supporting Materials and Methods).

## Results

### Aberrant expression of Lnc34a in human HCC

**Fig. 1:**
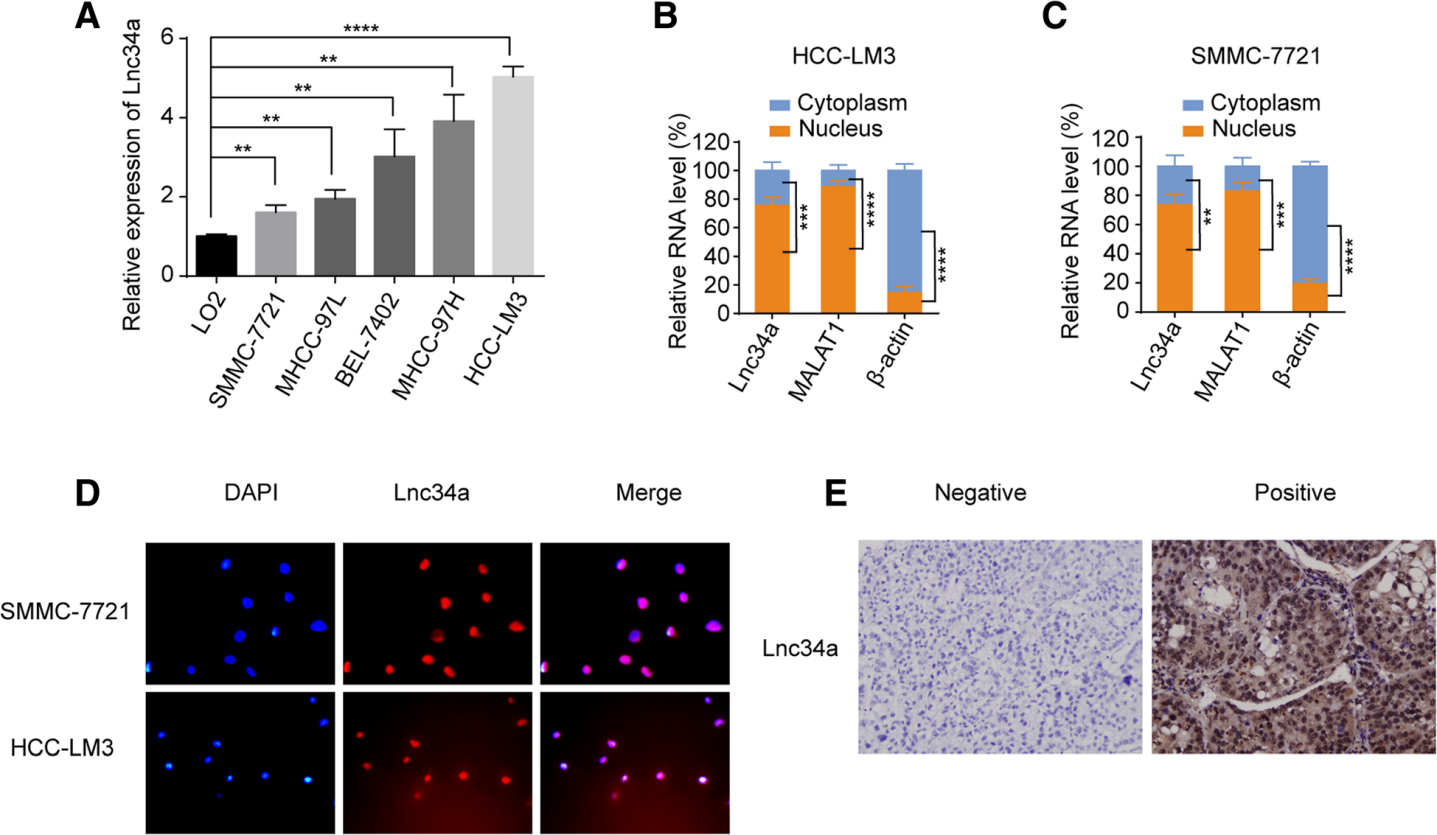
The aberrant expression of Lnc34a in human HCC. **a** Expression of Lnc34a in HCC cell lines and the immortalized human normal hepatocyte L02 cell line was detected by qRT-PCR and normalized to β-actin (*n* = 3). **b** and **c** The location of Lnc34a in SMMC-7721 and HCC-LM3 cells was identified by conducting a subcellular fractionation assay and qRT-PCR (*n* = 3). MALAT1 and β-actin were used as the nuclear and cytoplasm controls, respectively. **d** Lnc34a expression in SMMC-7721 and HCC-LM3 cells was detected by RNA-FISH (Magnification: × 200). **e** The typical images of Lnc34a expression in hepatocellular carcinoma tissue microarrays. Magnification: × 200. **P < 0.01; ***P < 0.001; ****P < 0.0001

To examine the relative expression of Lnc34a in the immortalized normal human hepatocyte cell line L02 and a serious of HCC cells with different metastatic potential, qRT-PCR was performed. Compared to the immortalized human normal hepatocyte L02 cell line, five types of HCC cell lines showed higher Lnc34a expression levels (Fig. 1a). Subcellular fractionation assays showed that Lnc34a was mainly in the nuclear of HCC-LM3 cell (Fig. 1b) and SMMC-7721 cell (Fig. 1c), and RNA fluorescence in situ hybridization (RNA FISH) also suggest that Lnc34a was primarily located in the nucleus (Fig. 1d); thus, Lnc34a may function via epigenetic regulation.

To define the role of Lnc34a in human HCC BM, we measured Lnc34a expression level by using ISH based on intratumoral TMAs from 252 HCC patients. Lnc34a was mainly located in the nuclei of tumor cells, with representation in cytoplasm (Fig. 1e). Intratumoral Lnc34a was positively expressed in 121 (48.0%) patients. The associations between lnc34a expression in tissue and clinicopathologic factors were analyzed (Additional file 2: Table S1). As shown in Table 1, univariate analysis showed that tumor encapsulation, vascular invasion, BCLC stage, and intratumoral Lnc34a were significantly correlated with BM. Multivariate Cox proportional hazards analyses revealed that vascular invasion, BCLC stage, and intratumoral Lnc34a were independent prognostic factors for developing BM in HCC patients (Additional file 3: Table S2).

**Table 1:** Univariate analyses of factors associated with bone metastasis in 252 HCC patients

Variable	Bone metastasis	
	HR (95% CI)	*P*
Age (≤51 versus > 51 years)	1.021 (0.520–2.004)	0.952
Gender (male versus female)	1.109 (0.429–2.866)	0.830
HBsAg (negative versus positive)	0.735 (0.333–1.625)	0.447
HCV-Ab (negative versus positive)	1.608 (0.219–11.829)	0.641
AFP, ng/mL (≤ 20 versus > 20)	0.597 (0.295–1.206)	0.150
ALT,U/L (≤ 40 versus > 40)	1.356 (0.648–2.836)	0.419
γ-GT,U/L (≤ 50 versus > 50)	1.275 (0.650–2.501)	0.480
Liver cirrhosis (no versus yes)	1.039 (0.430–2.510)	0.933
Child-Pugh score (A versus B)	2.714 (0.650–11.336)	0.171
Tumor differentiation (I–II versus III–IV)	0.464 (0.163–1.317)	0.149
Tumor size, cm (≤ 5 versus > 5)	0.716 (0.358–1.430)	0.344
Tumor number (single versus multiple)	1.108 (0.482–2.544)	0.810
Tumor encapsulation (complete versus none)	4.112 (1.791–9.444)	0.001
Vascular invasion (no versus yes)	13.116 (6.379–26.969)	< 0.001
BCLC stage (0-A versus B-C)	18.099 (8.894–36.834)	< 0.001
Lnc34a (negative versus positive)	9.780 (3.440–27.806)	< 0.001

*HCC* Hepatocellular carcinoma, *HBsAg* Hepatitis B surface antigen, *HCV-Ab* Hepatitis C virus antibody, *AFP* A-fetoprotein, *ALT* Alanine aminotransferase, *γ-GT* γ-glutamyl transferase, *BCLC-stage* Barcelona clinic liver cancer-stage

### Lnc34a promotes the proliferation and motility of hepatoma cells and BM

**Fig. 2:**
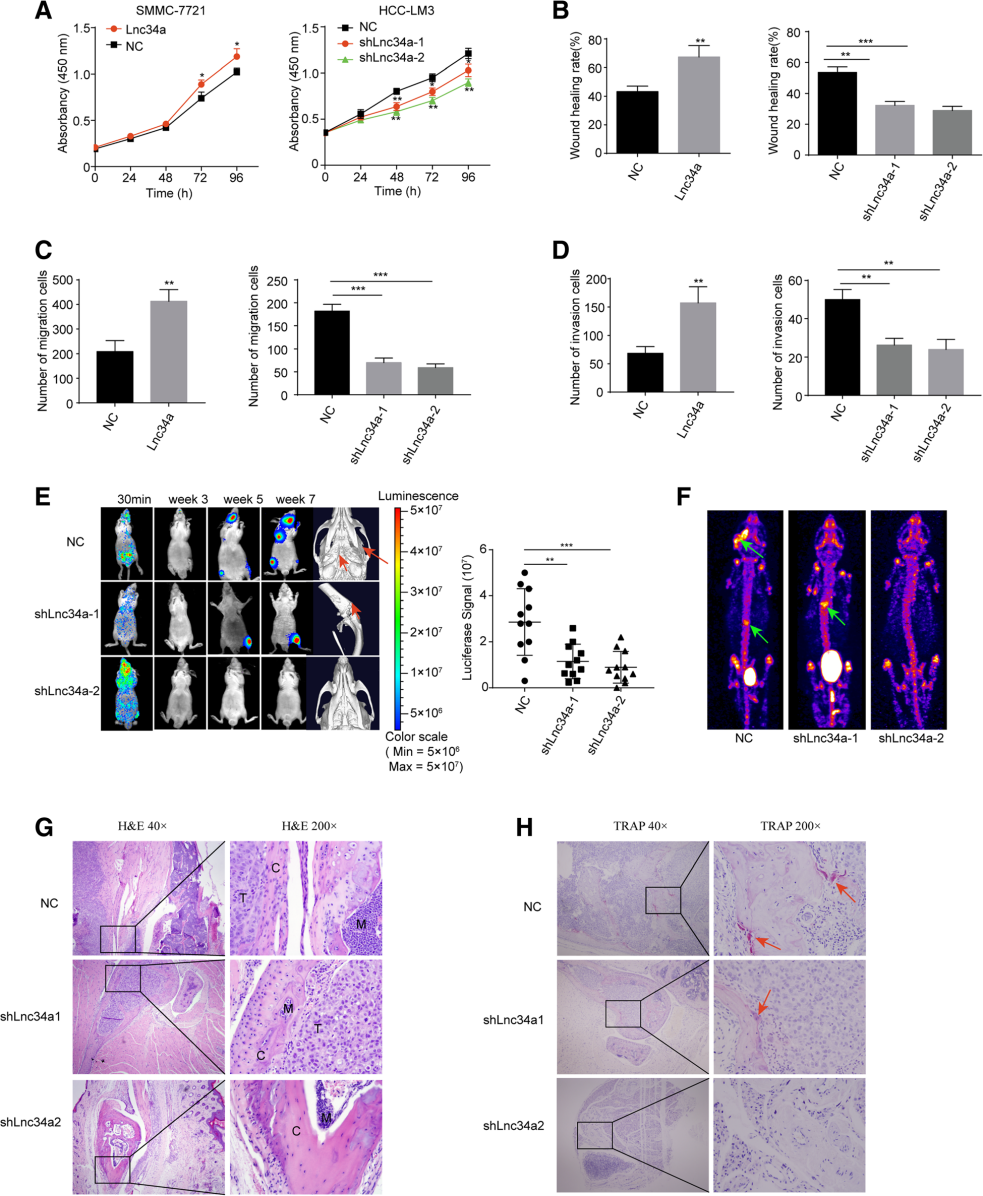
Lnc34a promoted the proliferative and motile ability of hepatoma cells in vitro and BM in vivo. **a** Effect of Lnc34a on cell proliferation was measured by CCK8 (*n* = 3). The wound-healing assay (**b** Magnification × 40) and transwell assay (Magnification × 100) without (**c**) or with Matrigel (**d**) were performed to analyze the effect of Lnc34a on the migration and invasion of SMMC-7721 and HCC-LM3 cells. **e** Bioluminescent imaging (BLI) and micro-CT of bone metastases by HCC-LM3 cells. The red arrowheads denote areas of overt osteolysis. BLI quantitation of metastasis by HCC-LM3 cells 7 weeks following an intracardiac injection (*n* = 11). **f** The representative graphs of SPECT bone scanning at seventh week of mice with metastases in mandible and vertebra. The site of bone metastasis was indicated by increased uptake of radiotracer (^99m^Tc-MDP), with characteristic radioactivity accumulation in the joints and metabolism of urinary system including bladder. **g** Representative H&E-stained sections (T, tumor; C, cortical bone; M, normal bone marrow). **h** Osteoclast TRAP staining. NC, negative control. *P < 0.05; **P < 0.01; ***P < 0.001; ****P < 0.0001

To explore the pathophysiological significance of Lnc34a in HCC, we further determined the effects of Lnc34a on the abilities of in vitro proliferation, migration, and invasion of HCC cells. SMMC-7721 cell which had lower intrinsic Lnc34a level was transfected with Lnc34a overexpression lentiviral vector to upregulate its Lnc34a expression, while the HCC-LM3 cell with the high-intrinsic Lnc34a was transfected with shRNAlnc34a-1 or shRNAlnc34a-2 lentiviral vectors to down-regulate its Lnc34a level. Expression of Lnc34a was verified by qRT-PCR (Additional file 4: Figure S1A). The up-regulation of Lnc34a in SMMC-7721 cell promoted its proliferation, whereas down-expression of Lnc34a in HCC-LM3 cell resulted in significant suppression of cell proliferation (Fig. 2a). The migration capability of SMMC-7721 cells in wound healing assays was significantly increased after transfection of Lnc34a (Fig. 2b; Additional file 4: Figure S1B). In contrast, knockdown of Lnc34a decreased wound healing (Fig. 2b; Additional file 4: Figure S1B). Similarly, in transwell assays with or without Matrigel, SMMC-7721 cell overexpressing Lnc34a displayed significantly higher migration and invasion abilities compared with control cells while the opposite results were observed in HCC-LM3 cell underexpressing Lnc34a (Fig. 2c and d; Additional file 4: Figure S1C and D). These findings suggest that Lnc34a promoted HCC cell migration and invasion in vitro.

To further determine the effects of Lnc34a on BM in vivo, luciferase-labeled HCC-LM3 cells stably down-expressing Lnc34a or control vector were injected into the left ventricle of nude mice. Luciferase signals were detected using ex vivo imaging 30 min, 3 weeks, 5 weeks and 7 weeks after injection. The success of intracardiac injections was identified by the distributing luminescence signal throughout the body of the mice 30 min after injection (Fig. 2e). Furthermore, micro-computed tomography (micro-CT) scan indicated that cancer cell-induced osteolysis was repressed in the shLnc34a groups (Fig. 2e). In mice of the NC group, the formation of metastatic lesions was first detected on day 21 after inoculation. Statistical analysis showed significantly lower luciferase signal in nude mice inoculated with luciferase-labeled HCC-LM3 cells stably down-expressing Lnc34a by shLnc34a-1 or shLnc34a-2 than in mice injected with NC (Fig. 2e). The representative graphs of SPECT were shown in Fig. 2f, with characteristic radioactivity accumulation in the joints and metabolism of urinary system including bladder. ^99m^Tc-MDP was obviously accumulated in the mandible and lumbar vertebrae. Furthermore, micro-CT scan visually indicated that cancer cell-induced osteolysis was repressed in the shLnc34a group (Fig. 2e). And 45.5% (5 of 11) of mice injected with NC showed BM after 7 weeks by micro-CT imaging, including metastases to the skull, scapula, spine, and tibia. However, BM were identified in 18.2% (2 of 11) of mice injected with HCC-LM3 cells expressing shLnc34a-1 or 9.1% (1 of 11) of mice injected with HCC-LM3 cells expressing shLnc34a-2 7 weeks after inoculation. Histological sections with H&E staining clearly showed metastatic tumour cells in the mandible and the tibia, with destruction of normal bone tissue (Fig. 2g). TRAP staining showed that both the number and activity of osteoclasts were markedly reduced at the boundary in the shLnc34a groups (Fig. 2h).

### Lnc34a suppresses miR-34a expression

**Fig. 3:**
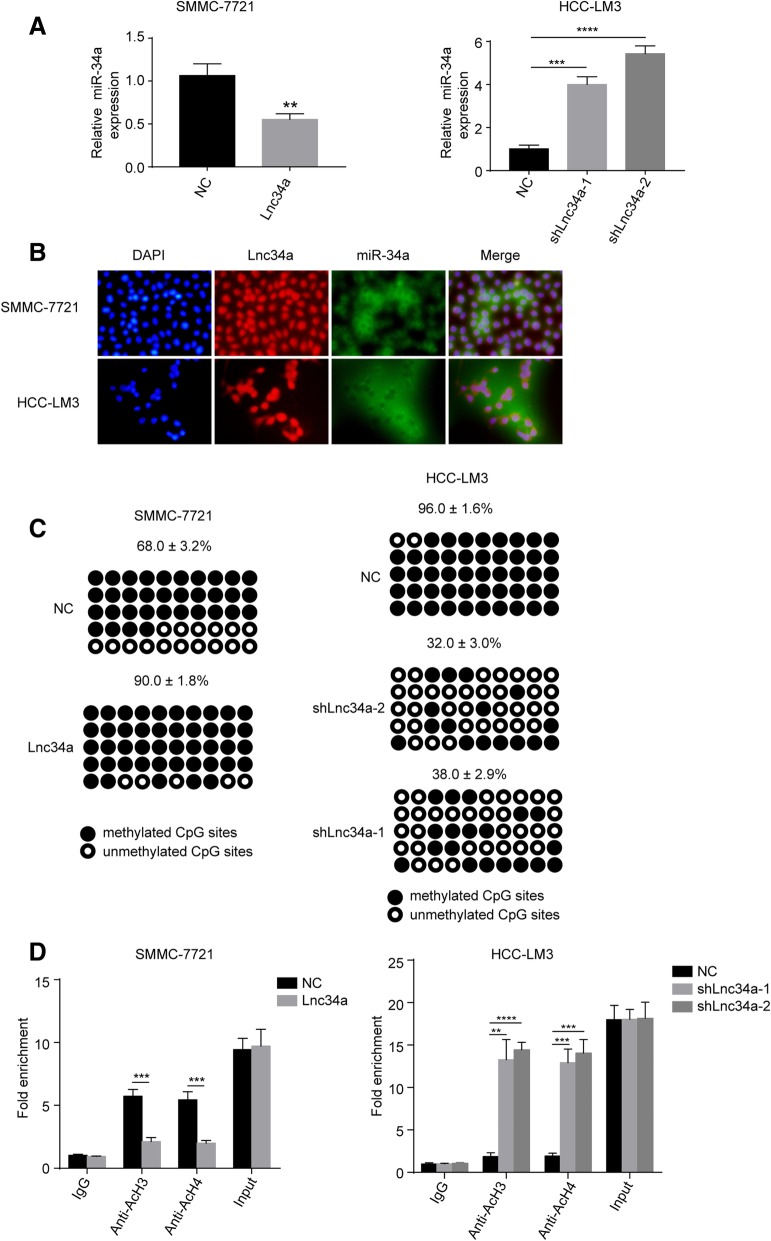
Lnc34a suppresses miR-34a expression by miR-34a promoter methylating and histone deacetylating (*n* = 3). **a** Ectopic Lnc34a suppresses miR-34a expression in SMMC-7721 cells and knock-down of Lnc34a increases miR-34a levels in HCC-LM3 cells. **b** The location of Lnc34a and miR-34a in SMMC-7721 and HCC-LM3 cells, as determined by RNA-FISH (Magnification: × 200). **c** Bisulfate-sequencing PCR results showing that the knock-down of Lnc34a decreased methylation on the miR-34a promoter in HCC-LM3 cells, while the over-expression of Lnc34a induced the hypermethylation of the miR-34a promoter. **d** The association between the acetylated histones H3 or H4 and the miR-34a promoter was decreased in SMMC-7721 cells with the Lnc34a ectopic expression using ChIP-qPCR with antibodies against the acetylated histones H3 and H4, whereas the opposite result was found in HCC-LM3 cells with the knockdown expression of Lnc34a. **P < 0.01; ***P < 0.001; ****P < 0.0001

To explore its molecular mechanism, we next found that the up-regulation of Lnc34a decreased miR-34a expression in SMMC-7721 cells (*P* < 0.01) (Fig. 3a). In contrast, knockdown of Lnc34a could significantly increase miR-34a levels in HCC-LM3 cell (*P* < 0.01) (Fig. 3a). Moreover, there was a significant negative correlation between Lnc34a and miR-34a expression in human HCC tissues examined by ISH with a correlation coefficient of *r* = − 0.326. These findings suggest that Lnc34a may promote BM at least partially through inhibiting miR-34a expression in HCCs. RNA FISH further revealed that Lnc34a and miR-34a were mostly present in opposite cell compartments in HCC-LM3 cells and SMMC-7721 cells (Fig. 3b).

Wang et. al has reported that Lnc34a repressed miR-34a expression by promoting its promoter methylation and histone deacetylation [18]. Up-regulation of Lnc34a enhanced miR-34a promoter methylation in SMMC-7721 cell, whereas decreased Lnc34a expression significantly diminished miR-34a promoter methylation, compared with the respective control groups (Fig. 3c). Moreover, ChIP-qPCR showed that increased expression of Lnc34a decreased associations between the acetylated histones H3 and H4 and the miR-34a promoter, whereas Lnc34a knockdown increases this combination (Fig. 3d). Therefore, Lnc34a repressed miR-34a expression in HCC cells by promoting the miR-34a promoter methylation and histone deacetylation in HCC cells.

### Lnc34a interacts with epigenetic regulators

**Fig. 4:**
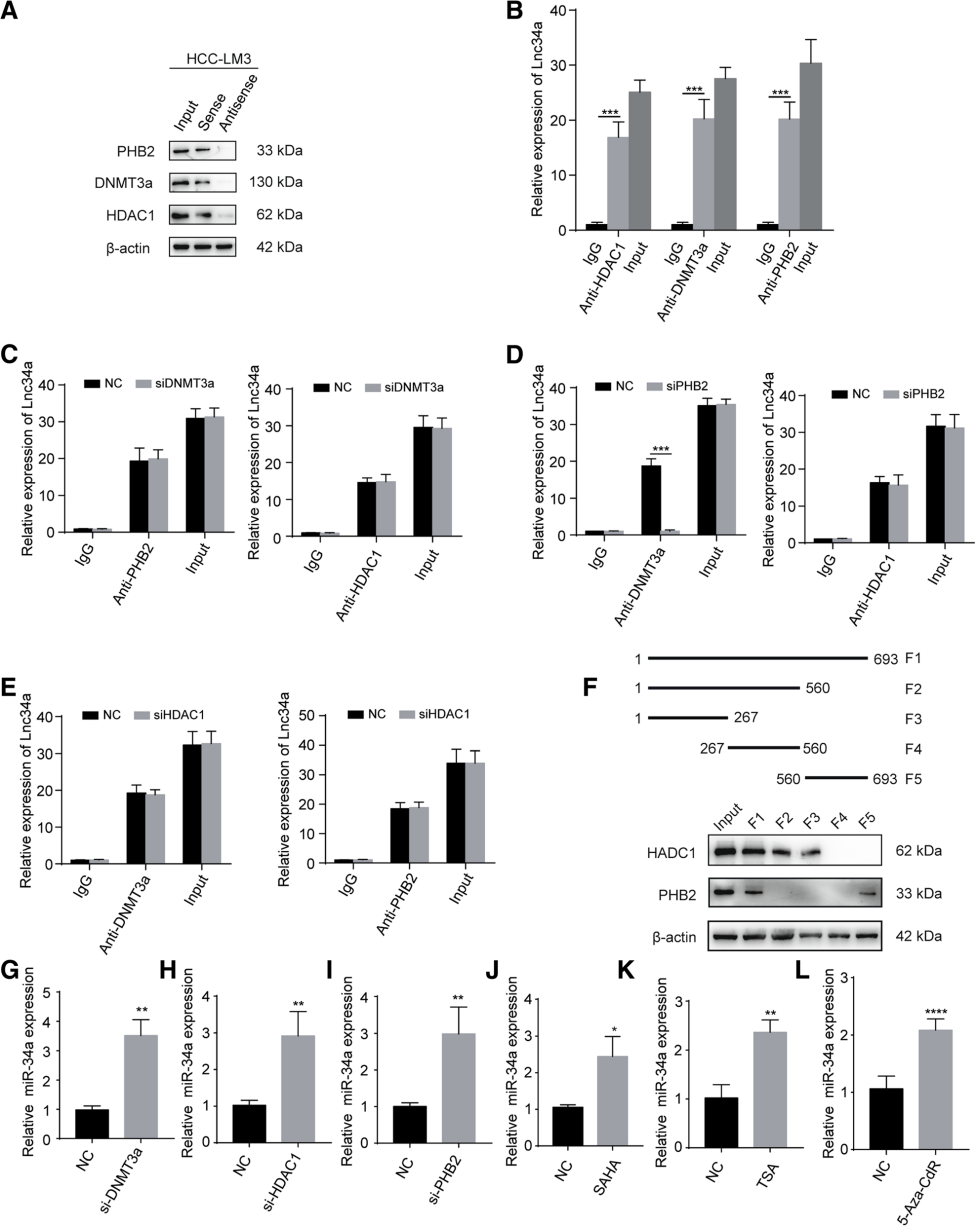
Lnc34a interacts with epigenetic regulators (*n* = 3). The interactions between Lnc34a and PHB2, DNMT3a, and HDAC1 were assessed by western blot following an RNA pull-down (**a**) and RIP assays (**b**) with HCC-LM3 cell. **c** RIP showing that the silencing of DNMT3a does not impact the interaction between Lnc34a with PHB2 or HDAC1. **d** RIP showing that the silencing of PHB2 disrupts the interaction between Lnc34a and DNMT3a, but has no effect on the interaction of Lnc34a with HDAC1. **e** RIP showing that the silencing HDAC1 has almost no effect on the interaction between Lnc34a with DNMT3a and a rare effect on the interaction of Lnc34a with PHB2. **f** PHB2 and HDAC1 were examined by western blot following RNA pull-down in samples with the different Lnc34a probe fragments and input samples. **g-i** Silencing the expression of DNMT3a (**g**), HDAC1 (**h**), and PHB2 (**i**) increased miR-34a expression levels in HCC-LM3 cells. **j**-**l** The expression level of miR34a was examined by qRT-PCR in HCC-LM3 cells treated with HDAC inhibitor SAHA (**j**) or TSA (**k**) and DNA methylation inhibitor 5-Aza-CdR (**l**). Three independent experiments were performed in the above assays. *P < 0.05; **P < 0.01; ***P < 0.001; ****P < 0.0001

We next explored whether Lnc34a regulates miR-34a expression through epigenetic regulators (i.e., DNMT3a, HDAC1, and PHB2) based on a literature review [18]. The association between Lnc34a and DNMT3a, HDAC1, and PHB2 was determined using an RNA pull-down assay and RIP assay in HCC-LM3 cell with high lnc34a expression (Fig. 4a and b). RNA pull down assay indicated that DNMT3a, HDAC1, and PHB2 were identified in the proteins pulled down by lnc34a probe (Fig. 4a). And there was lnc34a in the nucleic acids associated with DNMT3a, HDAC1 and PHB2 (Fig. 4b).

We further investigated how Lnc34a interacts with DNMT3a, HDAC1, and PHB2 using an RIP assay. While silencing DNMT3a did not affect the binding of Lnc34a with either PHB2 or HDAC1 (Fig. 4c), silencing PHB2 inhibited the interaction between Lnc34a and DNMT3a but did not prevent Lnc34a from binding to HDAC1 (Fig. 4d). In addition, HDAC1 silencing did not block the interaction between Lnc34a and DNMT3a or PHB2 (Fig. 4e). Together these data indicated that Lnc34a interacted with PHB2 and HDAC1, and recruited DNMT3a via PHB2.

We next examined the association between epigenetic regulators with serially truncated Lnc34a probes using an RNA pull-down assay. We found that the 1–267 bp fragment was sufficient to bind to HDAC1, and the 560–693 bp fragment was sufficient to bind PHB2 (Fig. 4f). Therefore, Lnc34a recruits HDAC1 and DNMT3a via PHB2 at least partly through the 1–267 bp and 560–693 bp sequences and then affects the miR-34a promoter to regulate miR-34a expression. However, whether Lnc34a binds these proteins through its specific structure needs to further explored.

We then silenced DNMT3a, HDAC1, and PHB2, respectively, and examined miR-34a expression by qRT-PCR. Decreased expression of DNMT3a, HDAC1 or PHB2, increased miR-34a levels (Fig. 4g-i). And inhibition of HDAC1 activity by Suberoylanilide Hydroxamic Acid (SAHA, Fig. 4j) or Trichostatin A (TSA, Fig. 4k), and DNA methylation inhibition by 5-aza-2′-deoxycytidine (5-Aza-CdR) also upregulated miR-34a expression (Fig. 4l). The results described above indicate that the level of miR-34a expression was influenced by these epigenetic regulators.

### MiR-34a inhibits TGF-β1-stimulated target gene expression and the motility of hepatoma cells

Our previous studies suggest that the intratumoral expression of both CTGF and IL-11 was associated with a higher incidence of BM in HCC [7]. IL-11 and CTGF have both been implicated in BM and are TGF-β1-inducible genes in breast cancer cells [19]. To investigate whether a similar effect is induced in HCC cells, we treated SMMC-7721 and HCC-LM3 cells with different concentrations of TGF-β1 ranging from 0 to 20 ng/ml. In addition, the relative level of IL-11 and CTGF mRNA expression was maximized when the concentration of TGF-β1 was 10 ng/ml (Additional file 5: Figure S2A and B). Consistent with previous studies in breast cancer, the induction of IL-11 by TGF-β1 was rapid and significantly elevated, peaking at 2 h (Additional file 5: Figure S2C). Moreover, CTGF, another TGF-β1 responsive gene, was slightly increased (Additional file 5: Figure S2D).

**Fig. 5:**
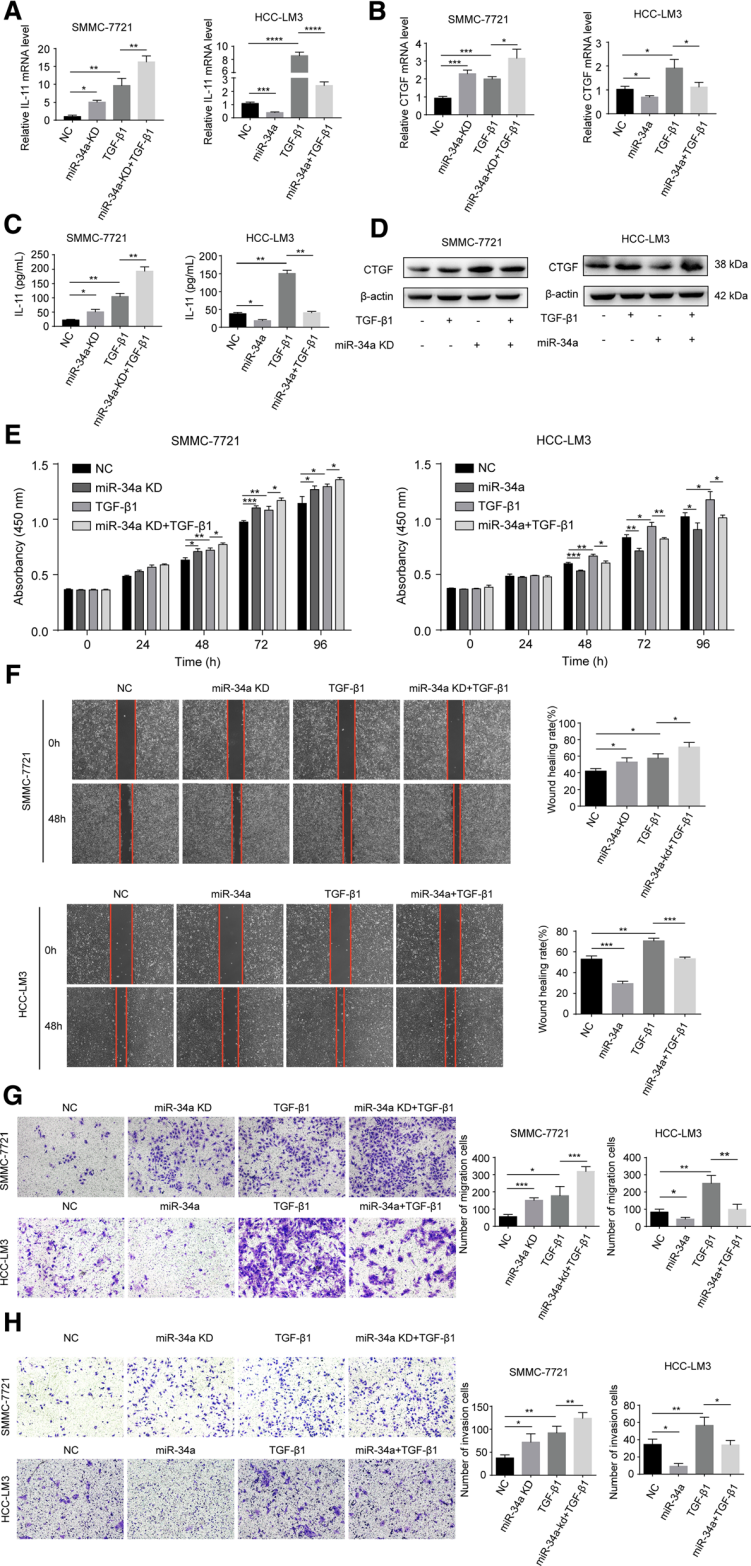
miR-34a inhibits TGF-β1-stimulated target gene expression and the proliferation and motility of hepatoma cells (*n* = 3). **a**-**d** miR-34a suppresses IL-11 and CTGF expression induced by TGF-β1 at both the mRNA (**a** and **b**) and protein level (**c** and **d**). The CCK-8 assay (**e**), wound-healing assay (**f** magnification × 40), and transwell assay (magnification: × 100) without (**g**) or with (**h**) Matrigel were performed to analyze the effect of miR-34a on the migration and invasion of SMMC-7721 and HCC-LM3 cells induced without or with TGF-β1. NC, negative control. *P < 0.05; **P < 0.01; ***P < 0.001; ****P < 0.0001

To assess the role of miR-34a on TGF-β1-induced genes, we transfected miR-34a or an anti-miR-34a viral vector into human HCC cell lines. The relative level of miR-34a expression was verified by qRT-PCR (Additional file 5: Figure S2E, F). As shown in Fig. 5a-d, the overexpression of miR-34a reduced the levels of IL-11 and CTGF mRNA and protein expression and inhibited TGF-β1-induced effects. In contrast, the knockdown of miR-34a expression led to an increased expression of the downstream targets and enhanced the effects of TGF-β1.

We next examined the effect of miR-34a on the TGF-β1-mediated migration, invasion, and proliferation of HCC cells. As a result, HCC-LM3 cells overexpressing miR-34a displayed significantly lower proliferation, migration, and invasion capabilities compared with the control cells and reduced TGF-β1-induced effects. The opposite results were observed in SMMC-7721 cells under-expressing miR-34a (Fig. 5e-h).

### Smad4 is a novel direct target of miR-34a in HCC cells

**Fig. 6:**
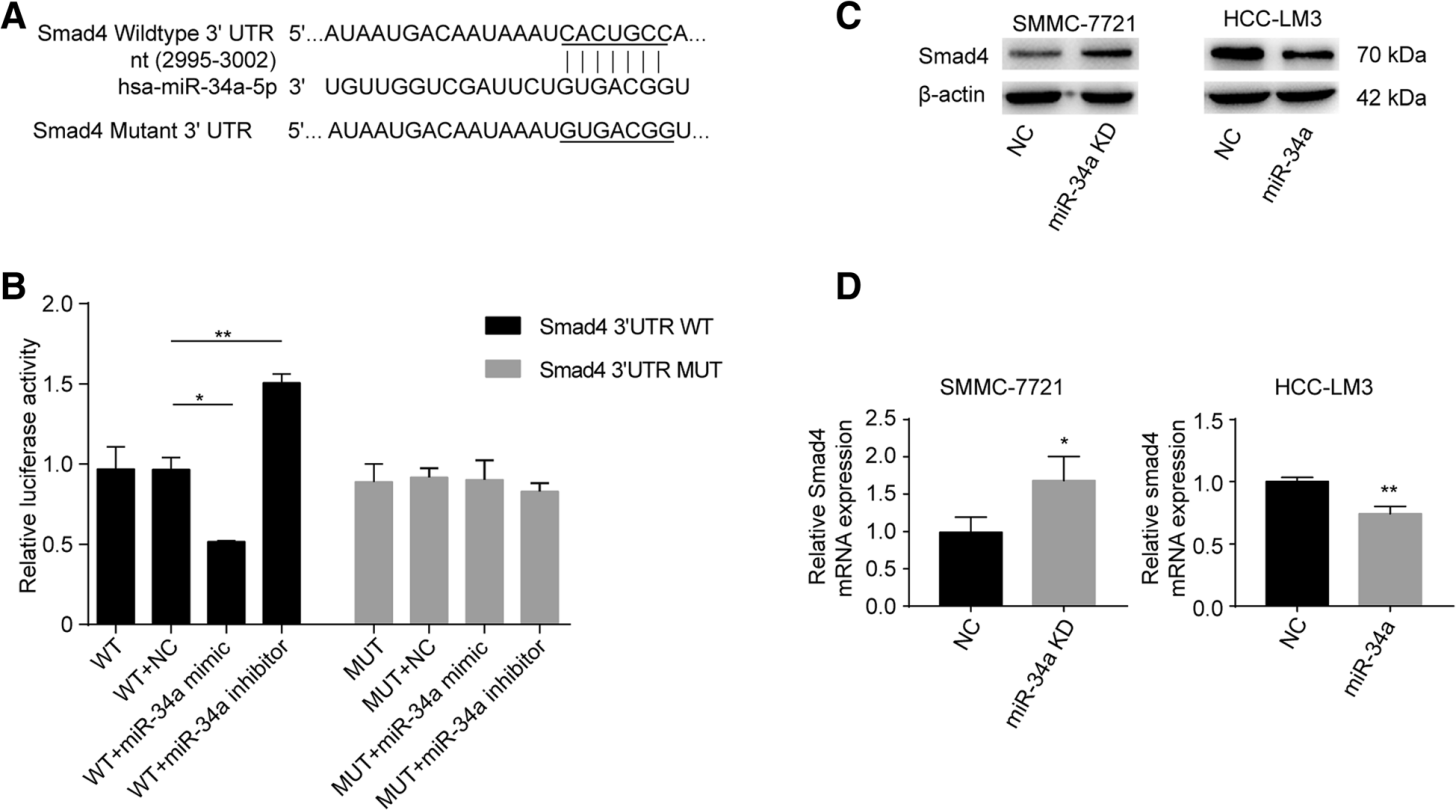
Smad4 is a direct downstream target of miR-34a (*n* = 3). **a** The target sites of miR-34a in the 3′-UTR of Smad4 were shown as a schematic representation predicted by TargetScan. The normal seed sequence and mutant were highlighted and underlined. **b** Luciferase activity with wild-type or mutant Smad4 3′-UTR was performed after co-transfection with the miR-34a mimic or inhibitor or respective controls into HEK293T cells. **c** and **d** The levels of Smad4 mRNA and protein expression in SMMC-7721 cells with knocked-down miR-34a expression or its negative control, and in HCC-LM3 cells overexpressing miR-34a or its negative control using western blot and qRT-PCR. NC, negative control. WT, wild-type. *P < 0.05; **P < 0.01

We then searched for candidate downstream targets of miR-34a using publicly available databases (i.e., miRwalk, miRanda, and Targetscan databases). Based on the above findings and literature review, Smad4 was selected for further experimental validation. Figure 6a indicates that the miR-34a binding sites were complementary to the 3′-UTR of Smad4 by a Targetscan analysis. To verify this prediction, we constructed plasmids with a firefly and renilla reporter containing either the wild type or mutant 3′-UTR of Smad4. While miR-34a was found to significantly suppress the luciferase activity of Smad4 with the wild type 3′-UTR, the mutant 3′-UTR did not suppress luciferase activity (Fig. 6b). The downregulation of miR-34a increased the luciferase activity of Smad4 containing the wild type 3′-UTR, whereas the luciferase activity with the mutant 3′-UTR remained virtually unaffected compared with the respective controls (Fig. 6b).

Moreover, the overexpression of miR-34a reduced the level of both Smad4 mRNA and protein expression in HCC-LM3 cells (Fig. 6c and d). In contrast, the levels of Smad4 mRNA and protein expression were increased in SMMC-7721 cells following the knockdown of miR-34a (Fig. 6c and d). Together, these findings indicate that Smad4 is a downstream target of miR-34a.

### MiR-34a targets Smad4 to inhibit TGF-β1-induced expression and migration of hepatoma cells

To further demonstrate that Smad4 is a functional target gene of miR-34a, we transfected SMMC-7721 cells with stable shSmad4 and HCC-LM3 cells with stable Smad4 (Additional file 6: Figure S3A and B). The analysis of TGF-β1-induced targeted expression revealed that the induction of IL-11 and CTGF mRNA and protein expression was attenuated in SMMC-7721 cells with shSmad4–2 compared with the control, whereas the opposite results were found in Smad4-overexpressing HCC-LM3 cells (Additional file 6: Figure S3C-F). Therefore, Smad4 is required for the activation of IL-11 and CTGF by TGF-β1. We then restored Smad4 expression in HCC-LM3 cells by up-regulating miR-34a expression under TGF-β1 stimulation. As expected, Smad4 markedly rescued the reduction of TGF-β1-responsive targets caused by restored miR-34a expression in the transfected HCC-LM3 cells compared with the respective controls. The opposite results were observed in SMMC-7721 cells (Additional file 6: Figure S3C-F).

We further examined the effect of Smad4 on the cellular migration, invasion, and proliferation of HCC cells. A knockdown of Smad4 expression in SMMC-7721 cells decreased the TGF-β1-induced migration, invasion, and proliferation capability compared with the control cells, whereas the opposite results were observed in HCC-LM3 cells with Smad4 overexpression (Additional file 6: Figure S3G-J). Thus, TGF-β1 promoted cellular migration, invasion, and proliferation in a Smad4-dependent manner. In the presence of TGF-β1, the knockdown of Smad4 reduced the cellular migration, invasion, and proliferation induced by the down-regulated expression of miR-34a in SMMC-7721 cells (Additional file 6: Figure S3G-J). In addition, Smad4 restored the inhibitory effects caused by miR-34a over-expression in HCC-LM3 cells following the induction of TGF-β.

### MiR-34a inhibits BM of HCC both in vitro and in vivo

**Fig. 7:**
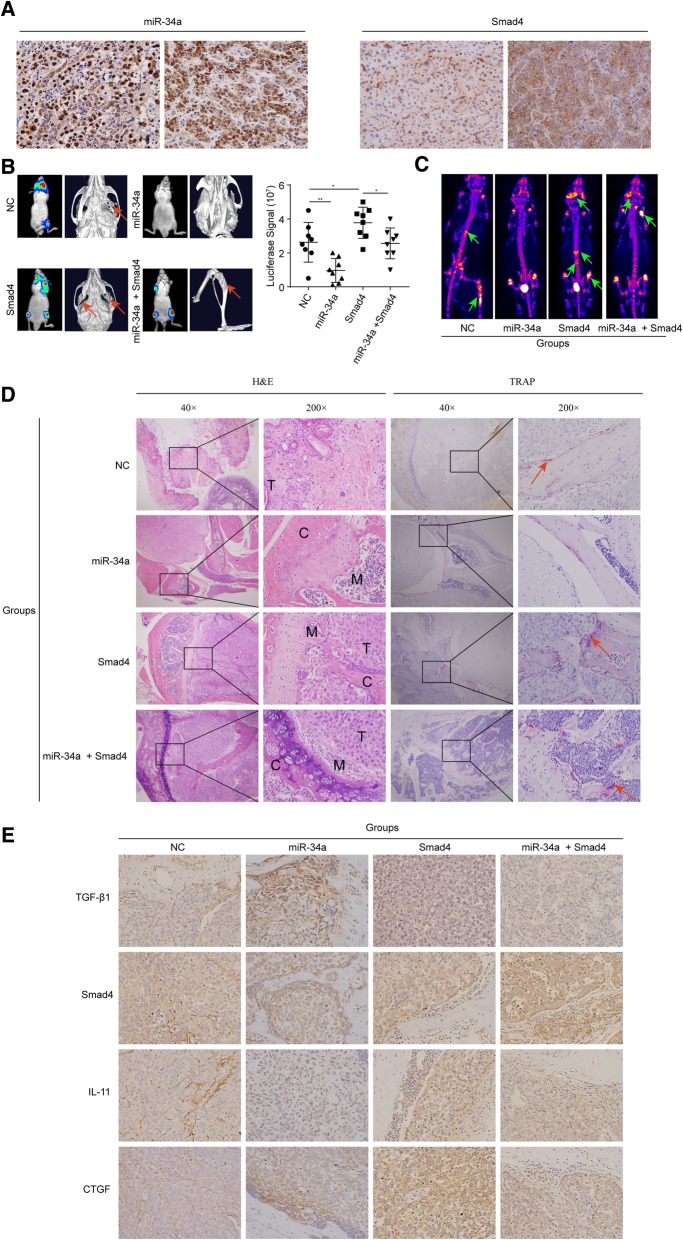
miRNA-34a inhibits BM in HCC both in vitro and in vivo. **a** Typical images of miR-34a by ISH and Smad4 by IHC. Magnification: × 200. **b** BLI and micro-CT of BM by HCC-LM3 cells transfected with miR-34a, Smad4, miR-34a plus Smad4 or NC. Arrowheads denote areas of overt osteolysis. BLI quantitation of metastasis 7 weeks after intracardiac injection (*n* = 8). **c** The representative graphs of SPECT bone scanning at seventh week of mice with metastases in tibia, mandible and lumbar vertebrae, indicated by increased accumulation of ^99m^Tc-MDP. **d** and **e** Representative H&E images, osteoclast TRAP staining, and IHC analyses of BM genes (Magnification: × 200). Arrows point to TRAP+ osteoclasts along the tumor-bone interface. NC, negative control. *P < 0.05; **P < 0.01

To further investigate the clinical relevance of Smad4 and miR-34a, the expression of Smad4 and miR-34a were analyzed by IHC and ISH, respectively. Both miR-34a and Smad4 were localized in the cytoplasm and nuclei of the tumor cells (Fig. 7a). Intratumoral miR-34a and Smad4 were expressed in 186 (73.8%) and 90(35.7%) patients, respectively. The evaluation scores of miR-34a and Smad4 were analyzed using a Spearman correlation analysis. As a result, the level of miR-34a expression was found to be inversely correlated with the level of Smad4 protein in HCC tissues (*r* = − 0.411).

We next analyzed the consequences of miR-34a, Smad4, or miR-34a plus Smad4 overexpression on HCC BM in vivo. HCC-LM3 cells overexpressing miR-34a or Smad4, co-expressing miR-34a/Smad4, or expressing the control vector were intracardially injected into the circulation of nude mice followed by BLI. BLI quantitation was shown in Fig. 7b. The representative graphs of SPECT were shown in Fig. 7c with ^99m^Tc-MDP obvious accumulation in the hind limb, mandible, lumbar vertebrae and scapula. After the animals were sacrificed, bone specimens from the vertebra, skull, and tibias were scanned via micro-CT. As a result, miR-34a overexpression was found to significantly inhibit BM, whereas Smad4 overexpression significantly accelerated the invasion of cancer cells into the bone and caused more severe bone damage (Fig. 7b). More importantly, Smad4 could partially reverse the inhibitory effect of miR-34a on the BM of HCC cells in mice (Fig. 7b). Together, these findings demonstrate that the up-regulation of Smad4 reversed the function of miR-34a in the BM of HCC cells. Representative H&E images, osteoclast TRAP staining, and IHC analyses of BM genes are presented in Fig. 7d-e.

## Discussion

BM are a common occurrence in several advanced malignancies, including breast, prostate, and lung cancer [20, 21]. Once established in the bone, the patient prognosis is extremely poor [22]. Thus, it is necessary to understand the molecular mechanisms regulating the establishment, growth, and activity of tumors in bone. However, little is known of the molecular mechanisms in BM from HCC. In the present study, Lnc34a, which has been previously only reported in colon cancer stem cell asymmetric division [18], was found to be up-regulated in HCC cell lines compared with the immortalized human hepatocyte, L02 cell line. Moreover, the overexpression of Lnc34a in the intratumoral tissue was associated with an increased incidence of BM in HCC patients. Moreover, Lnc34a promotes HCC cellular proliferation, migration, and invasion in vitro, and the knockdown of Lnc34a could inhibit BM of HCC in vivo. Furthermore, Lnc34a was found to epigenetically silence miR-34a expression via the binding of its promoter to HDAC1 and DNMT3a, which was recruited through PHB2.

In addition, miR-34a has been documented to inhibit cancer development in many solid tumors, including breast, skin, and prostate cancer [23–25]. Consistent with these previous reports, we observed miR-34a to act as a tumor suppressor in HCC. The low levels of miR-34a in the intratumoral tissue were associated with an increased incidence of BM in HCC patients. Moreover, miR-34a was found to decrease HCC cell proliferation, migration, and invasion in vitro and inhibit BM in vivo. Previous studies have shown that miR-34a is involved in various pathophysiological processes through the TGF-β pathway in several cancers [25–28]. For instance, in HCC patients, elevated TGF-β activity associated with the persistent presence of hepatitis B virus (HBV) in the liver tissue, and was shown to downregulate miR-34a expression; this led to the enhanced production of the chemokine CCL22, which recruited regulatory T cells to facilitate immune escape. Therefore, HBV infection and activation of the TGF-β-miR-34a-CCL22 axis facilitates portal vein tumor thrombus development of HBV-positive HCC through the creation of an immune-subversive microenvironment [25]. Moreover, miR-34a mediates the resistance of oxaliplatin in colorectal cancer cells by inhibiting macroautophagy via the TGF-β/Smad4 pathway [26]. Additionally, miR-34a inhibits EMT in nasopharyngeal carcinoma by targeting Smad4 through the TGF-β/Smad pathway [27]. Consistent with this finding, our studies also suggest that Smad4 was a target of miR-34a in HCC.

Smad4 is a pivotal transducer of the TGF-β pathway and plays complex and contradictory roles during tumorigenesis [29]. Smad4 (also termed deleted in pancreatic carcinoma, locus 4 [DPC4]), was initially identified as a candidate tumor suppressor gene whose inactivation may play a role in pancreatic cancer [30]. Similarly, the loss of Smad4 promotes cancer cell growth and metastatic progression in both colorectal and prostate cancer [31, 32]. Interestingly, Smad4 was found to be overexpressed in most extrahepatic cholangiocarcinoma patients [33], whereas the inactivation of Smad4 was identified in intrahepatic cholangiocarcinoma [34], which might be due to tumor heterogeneity. Similar situations have also been shown to occur in human HCC. Yao et al. found that tumor tissues expressed less Smad4 mRNA and protein than the adjacent tissues, whereas Smad4 protein levels were higher in the tissues of HCC patients with viral hepatitis compared to those who were uninfected [35]. Consistent with previous studies [36, 37], our study also suggests that Smad4 promotes the malignant potential of HCC.

Accumulating evidence suggests that Smad4 is required for TGF-β downstream genes, including CTGF and IL-11, which are involved in BM in breast cancer [19, 38, 39]. Yuan et al. demonstrated that IL-11 was necessary to increase the colonization potential of HCC cells by lncRNA-ATB [40]. CTGF is a proinvasive and angiogenic factor that promotes the motility of breast cancer cells and stimulates the formation of osteolytic lesions in animals [41]. During the bone colonization process, as targets of TGF-β via the Smad pathway, CTGF and IL-11 are associated with angiogenesis and osteoclast recruitment [4]. Our previous studies suggest that both CTGF and IL-11 are independent risk factors for the skeletal involvement observed in HCC patients [7]. In the present study, we identified Smad4 as a new target gene of miR-34a in HCC, which is involved in cellular progression and BM by regulating TGF-β signaling.

Several in vivo models have been established to study cancer metastasis to bone, among which intracardiac and intratibial injections are the most commonly used [42, 43]. Compared with a local intratibial injection that severely damages the tibia, an intracardiac injection successfully recapitulates the process of BM by injecting cancer cells into the left ventricle, which then disseminate throughout the entire body and eventually develop into metastatic colonies in the bone and other organs [44]. BM models have been well-studied in other cancers, including breast [39] and prostate cancer [45], but due to its requirement for a highly-accurate injection into the left ventricle of mice, have only been reported in HCC once [46]. In the present study, we established a BM model using intracardiac injections. Interestingly, in patients, the most frequent metastatic sites affected by BM from HCC were found in the axial skeleton, particularly in the thoracic and lumbar vertebrae [4]. In contrast, in the xenograft metastasis model established by intracardiac inoculation, the cancer cells metastasize predominantly to the maxillo-facial region, followed by the hind limbs and spine. This may be due to the highly vascularized red marrow and features of the mandible. The abrupt directional changes in the vessels of the mandibular angle and mental foramen have been shown to reduce blood velocity, which created favorable conditions for the formation of cancer emboli [47].

## Conclusions

In conclusion, our results suggest for the first time, that Lnc34a is associated with BM in HCC and regulates miR-34a expression by regulating its promoter methylation and histones deacetylation. On the other hand, Smad4 was modulated by miR-34a via the TGF-β pathway, which was followed by alterations in the downstream genes, CTGF and IL-11, which are associated with BM. Therefore, targeting the multiple mechanisms described above may provide beneficial evidence to prevent BM and facilitate the development of more effective targets for therapeutic strategies in HCC.

## Additional files

Additional file 1: Supporting Materials and Methods. (DOC 118 kb)

Additional file 2: Relationship between lnc34a expression in tissue and clinicopathological characteristics. **Table S1.** Relationship between lnc34a expression in tissue and clinicopathological characteristics. (DOC 88 kb)

Additional file 3: Multivariate analyses of factors associated with bone metastasis in 252 HCC patients. **Table S2.** Multivariate analyses of factors associated with bone metastasis in 252 HCC patients. (DOC 30 kb)

Additional file 4: The transfection efficiency of Lnc34a and typtical figures of wound healing assays and transwell assays with or without Matrigel. **Figure S1**. The validation of Lnc34a or shLnc34a transfections in HCC cell lines (*n* = 3). The transfection efficiency of Lnc34a or shLnc34a was conducted in SMMC-7721 and HCC-LM3 cells using qRT-PCR (A). The wound-healing assay (B; Magnification: × 40) and transwell assay (Magnification: × 100) without (C) or with Matrigel (D) were performed to analyze the effect of Lnc34a on the migration and invasion of SMMC-7721 and HCC-LM3 cells. NC, negative control. **P < 0.01; ***P < 0.001; ****P < 0.001. (TIF 3.33 MB)

Additional file 5: The activation of IL-11 and CTGF by TGF-β in HCC cells. **Figure S2.** The activation of IL-11 and CTGF by TGF-β in HCC cells (*n* = 3). (A) SMMC-7721 and HCC-LM3 cells were treated with different amounts of TGF-β1 (approximately 0–20 ng/ml) for 2 h followed by qRT-PCR was used to detect the expression of IL-11 and CTGF; (B) IL-11 and CTGF expression was examined by qRT-PCR following treatment with 10 ng/ml TGF-β1 for the various time periods (0, 1, 2, 4, 6, 8, 12, and 24 h). Modulation of miR-34a expression in SMMC-7721 and HCC-LM3 cells. The different levels of miR-34a expression in transfected SMMC-7721 (E) and HCC-LM3 cells (F) were respectively confirmed by qRT-PCR. NC, negative control. *P < 0.05; **P < 0.01; ***P < 0.001; ****P < 0.001. (TIF 606 KB)

Additional file 6: MiR-34a targets Smad4 to inhibit TGF-β1-induced target expression and migration of hepatoma cells. **Figure S3.** MiR-34a targets Smad4 to inhibit TGF-β1-induced target expression and migration of hepatoma cells (*n* = 3). Validation of transfection expression in SMMC-7721 and HCC-LM3 cells (A and B). qRT-PCR (C and D), ELISA (E), Western blot (F), wound-healing assay (G; Magnification × 40) and transwell assay (Magnification: × 100) without (H) or with (I) Matrigel and CCK-8 assay (J), were conducted in SMMC-7721 cells transfected with miR-34a kd, shSmad4, miR-34a kd plus shSmad4 or NC, and in HCC-LM3 cells transfected with miR-34a, Smad4, miR-34a plus Smad4 or NC, induced with TGF-β1. NC, negative control. *P < 0.05; **P < 0.01; ***P < 0.001. (TIF 6.18 MB)

## Data Availability

The data used or analyzed during this study are included in this article and available from the corresponding author upon reasonable request.

## Abbreviations

3’UTR: 3’untranslated regions
CCK-8: Cell Counting Kit-8
ChIP: Chromatin immunoprecipitation
CTGF: Connective tissue growth factor
DAPI: 4′,6-diamidino-2-phenylindole, dihydrochloride
DIG: Digoxigenin
DMEM: Dulbecco’s Modified Eagle’s Media
DNMT3a: DNA methyltransferase 3a
DPC4: Deleted in pancreatic carcinoma, locus 4
ELISA: Enzyme-linked immunosorbent assay
EMT: Epithelial-to-mesenchymal transition
FBS: Fetal bovine serum
FISH: Fluorescence in situ hybridization;
H&E: Hematoxylin and eosin
HBV: Hepatitis B virus
HCC: Hepatocellular carcinoma
HDAC1: Histone Deacetylase1
IL-11: Interleukin 11
IP: Immunoprecipitation
ISH: In situ hybridization
LNA: Locked nucleic acid
Lnc34a: LncRNA-34a
lncRNAs: Long noncoding RNAs
Micro-CT: Micro-computed tomography
miR-34a: MiRNA-34a
PBS: Phosphate buffer saline
PHB2: Prohibitin 2
PMSF: Phenylmethanesulfonyl fluoride
PVDF: Polyvinylidene fluoride
qRT-PCR: Quantitative real-time polymerase chain reaction
RBP: RNA-Binding Proteins
RIP: RNA immunoprecipitation
ROI: Region of interest
SDS-PAGE: Sodium dodecyl sulfate polyacrylamide gel electrophoresis
SPF: Specific pathogen free
SSC: Saline sodium citrate
TGF-β: Transforming growth factor β
TMAs: Tissue microarrays
TRAP: Tartrate-resistant acid phosphatase
