# Supplementary material for: The molecular mechanism of LncRNA34a-mediated regulation of bone metastasis in hepatocellular carcinoma
Source: Mol Cancer. 2019 Jul 26;18:120. doi: 10.1186/s12943-019-1044-9 (PMC6659280; doi:10.1186/s12943-019-1044-9)
Supplement: Supplementary file 1 — Supporting Materials and Methods. (DOC 118 kb) [file 12943_2019_1044_MOESM1_ESM.doc]

**Patients and tissue specimens**

252 patients undergoing curative resection for HCC at the Liver Cancer Institute, Zhongshan Hospital, Fudan University were collected in training cohort. Patient samples were collected from June 2002 to September 2007. A whole body bone scan was arranged to exclude BM before surgery and BM was not found in in any of the patients at the time of surgery. Additional inclusion criterias were as described previously[1].

**Follow-up**

Follow-up was completed on and the median follow-up time was 56.3 months (range, 2.6-120.2 months). As described in our previous study, a annual bone scan was performed. Abdominal ultrasound examination, as well as laboratory tests were performed every three months. And Chest radiography was performed every 6 months for detecting pulmonary involvement. Once bone pain occured, bone scanning or magnetic resonance imaging was immediately performed. A diagnosis of BM was based on a prior history of HCC, clinical presentation, and radiological imaging tests. The time period from the date of surgery to the date of BM presentation was recorded. Treatments after recurrence was as follows: once BM occured, external beam radiotherapy was conducted for the involved bone; radiotherapy, interventional therapy, or surgery was used for other site relapses.

**TMA, Immunohistochemistry (IHC) and In situ hybridization (ISH)**

The experimental procedures for TMA, IHC and ISH were performed as previously described[1, 2]. The expression levels of Smad4 were scored using a previously described scoring system: score 0: 0-10% positive staining, score 1: 10-30% positive staining, score 2: 30-70% positive staining, score 3: 70% positive staining, score 4: 70% positive staining+high intensity. Then the following scores were applied: low, 0 ≤ score < 2; moderate, 2 ≤ final score < 3; high, 3≤ final score ≤ 4[3]. MiR-34a expression levels were scored using a semi-quantitative scoring system which combines the signal intensity (0 = no signal, 1 = weak signal, 2 = intermediate signal, and 3 = strong signal) and percentage of positive cells ( none = 0; <1% =1; 1–10% = 2; 10–33% = 3; 34–67% = 4; and > 67% = 5) to produce a score that ranges from 0–8. According to the final score, the expression signal was determined as negative (0-2), positive (3-8)[4]. For Lnc34a expression level, Image-Pro Plus version 6.0 software (Media Cybernetics, Inc., Rockville, MD, USA) was used to assess the area and integrated optical density (IOD) density of the chromogenic region. An identical setting was used for all images. The mean density of Lnc34a in each image was calculated as IOD/total area of each image. For Lnc34a density, the median value was used as the cutoff for the definition of subgroups[5].

**Cell lines**

The human HCC cell lines MHCC-97L, MHCC-97H, HCC-LM3 were obtained from the Liver Cancer Institute of Fudan University, and the immortalized human hepatocyte cell lines, L02, SMMC-7721, and BEL-7402 were purchased from the Shanghai Advanced Research Institute, Shanghai Chinese Academy of Sciences. These cells were cultuered in high glucose Dulbecco’s Modified Eagle Medium (DMEM) with 10% fetal bovine serum (FBS) and 1% penicillin G and streptomycin sulfate at 37C in a 5% CO2 humidified atmosphere. 5-Aza-CdR (Sigma, USA) was diluted in DMSO and used at 2 μM concentration. And cells were treated with 200 nM TSA and 5 μM SAHA (Santa Cruz, USA) for 72-96 h. And medium was replaced every 24 h.

**Cell transfections**

The lentivirus vectors for Lnc34a, shLnc34a, miR-34a, miR-34a KD, Smad4, shRNAs plasmids for Smad4 and corresponding negative control sequence were obtained from Genechem Company Ltd (Shanghai, China). The cells were plated in six-well plates and incubated with 1 ml viral supernatants in the presence of polybrene (5 μg/ml) for 8 hours. Cells were subsequently propagated in selection medium containing puromycin. And quantitative real-time PCR (qRT-PCR) and western blot were performed to detect the level of Lnc34a, miR-34a and Smad4 expression. The shRNA oligonucleotides were obtained from literature: shRNA targeted against Smad4 sequence GGATGAATATGTGCATGAC (Smad4-shRNA1) GGTGTGCAGTTGGAATGTA (Smad4 shRNA2) [6] and shRNA targeted against the Lnc34a sequence CCAGAGATTATGAAATCAAAG (Lnc34a-shRNA1); GGAGGCTACACA ATTGAACAG (Lnc34a-shRNA1) [7]. Specific shRNAs targeting DNMT3a, PHB2, and HDAC1 were purchased from Santa Cruz Biotechnology (Dallas, TX). Cellular transfection was performed by using Interferin siRNA transfection reagent (Polyplus transfection, Illkirch, France) according to the protocol.

The wild-type 3’untranslated regions (3’UTR) segment of human Smad4, which contained a putative binding site for miR-34a, was sub-cloned into the psi-CHECK2 vector (Promega, Madison, WI, USA) using XhoI and NotI restriction sites. The psi-check2 vector is a dual-luciferase reporter plasmid that contains the Firefly luciferase and Renilla luciferase reporter genes. The mutant 3'-UTR of Smad4 had a mutated sequence in the complementary site for the seed region of miR-34a. All constructs were verified by direct sequencing. Transfections were performed using jetPRIME® (Polyplus transfection, Illkirch, France).

**RNA isolation and qRT-PCR**

Total RNA were extracted from cell lines using TRIzol reagent (Invitrogen, California, USA). MiR-34a expression was measured using the Bulge-Loop™ miRNA qRT-PCR Starter Kit (RiboBio, Guangzhou, China) according to the manufacturer's instructions. As for the mRNA analysis of CTGF, Smad4, β-actin, IL-11 or Lnc34a, complementary DNA synthesis (cDNA) was synthesized using PrimeScript reverse transcriptase reagent kit (Takara Bio, Inc., Japan). Amplification and detection were performed by using SYBR Green PCR master mix (Takara, Tokyo, Japan) in the 7500 real-time PCR system (Applied Biosystems, Thermo Fisher Scientific, Waltham, MA,USA). β-actin or U6 was used as an endogenous control. The relative expression of miR-34a and other genes were normalized to U6 and β-actin, respectively, by using 2^−ΔΔCt^ method. The sequences of sense and antisense primers were as follows:

CTGF, 5’-ACCGACTGGAAGACACGTTTG-3’ and 5’-CCAGGTCAGCTTCGCAA GG-3’; IL-11, 5’-CGAGCGGACCTACTGTCCTA-3’and 5’-GCCCAGTCAAGTGT CAGGTG-3’; Smad4, 5’-CTCATGTGATCTATGCCCGTC-3’ and 5’-AGGTGATA CAACTCGTTCGTAGT-3’; β-actin, 5’-TGACGTGGACATCCGCAAAG-3’ and

5’-CTGGAAGGTGGACAGCGAGG-3’; MALAT1, 5’-CTGTGGTGTGGGATT GAGGC-3’ and 5’-GCAGGGACGGTTGAGAAGTG-3’ ; and Lnc34a, 5’-GGAGGC TACACAATTGAACAGG-3’ and 5’-AGTCCGTGCGAAAGTTTGC-3’.

**Subcellular fractionation**

The separation of the nuclear and cytosolic fractions was performed using a PARIS Kit (Life Technologies) according to the manufacturer's instructions. The RNAs in the nuclei and cytoplasm were isolated and extracted from the SMMC-7721 and HCC-LM3 cells, and the expression levels of Lnc34a in the nuclei and cytoplasm of both cells were examined by qRT-PCR. MALAT1 and β-actin were detected as fractionation indicators.

**RNA Fluorescence in Situ Hybridization (FISH)**

SMMC-7721 cell and HCC-LM3 cell were seeded into multi-chambered coverglass slides (Lab-Tek, Nalge Nunc, Rochester, New York, United States) and incubated overnight under normal growth conditions, reaching 50-70% confluency. Then the cells were fixed with 4% paraformaldehyde for 30 min at room temperature, washed three times with 1×PBS, and permeabilized with 0.1% Triton X-100 in PBS for 5 min at 4℃. After pre-hybridization at 37℃ for 30 min, the slides were incubated in a humid chamber with the Digoxigenin (DIG)–labeled locked nucleic acid (LNA) probe against Lnc34a (80 nM) or FAM-labeled probe against miR-34a (40 nM) for 1 h at 55°C for hybridization. Slides were then washed once with 5× saline sodium citrate (SSC) buffer, twice with 1× SSC buffer, followed by three washes with 0.2× SSC buffer at 55°C. Chromogenic detection of signals was performed using an anti-DIG secondary antibody conjugated with Alexa Fluor647 (Jackson, USA) for 1 h according to the manufacturer’s instructions. Nuclei were counterstained with 4’, 6-diamidino-2-phenylindole, dihydrochloride (DAPI, Invitrogen) and the images were captured using fluorescence microscopy (FV300, Olympus, Japan).

**Cell Proliferation**

Cell proliferation was determined by using Cell Counting Kit-8 (CCK-8, Dojindo, Kumamoto, Japan) according to the manufacturer's instructions. Brifely, the transfected cells with or without TGF‐β1(10 ng/ml; PeproTech) treatment were seeded at a density of 2,000 cells per well in 96-well plates, and cultured until 96 hours. Cell proliferation was assessed every 24 h. The absorbance at 450 nm was measuredusing a microplate reader after incubation for 2 hours at 37°C. All experiments were performed in triplicate and repeated at least three times.

**Wound healing assay**

Transfected cells were seeded in 6-well plates and reached full confluency overnight. The cell layer was scratched with 200 μl pipette tips and washed twice with phosphate buffer saline (PBS) to remove the floating cells. Then the cells were cultured with DMEM contained 1% FBS. The wound width was measured at 0, 24, and 48 hours by using a microscope (Olympus, Tokyo, Japan) at 40× magnification.

**Migration and invasion assay**

The migratory or invasive abilities of HCC cells were evaluated using 24-well transwell chambers coated with or without Matrigel (BD Pharmingen, San Jose, CA). Totally, 200 μl of the single cell suspension (2-5 × 10^4^ cells per well) were seeded in the upper chamber of the 8 μm-pore transwell filters (Costar, Lowell, MA, USA) with serum-free DMEM, and the lower chambers were supplemented with 600 μL of 10% FBS/DMEM containing or no TGF‐β1(10 ng/ml). After incubation for 48-72 h at 37 °C, a sterile cotton swab was used to remove the cells remaining in the upper upper membrane of the transwell chamber. Then the transwell chambers were fixed with 4% paraformaldehyde and stained with 0.1% crystal violet (Beyotime, Shanghai, China). The cells that migrated to the bottom side of the insert were photographed and counted in 5 randomly selected fields by using an inverted microscope (Olympus, Tokyo, Japan) at 100× magnification.

**Western blot analysis**

Total protein was extracted by lysing cells in RIPA buffer containing phenylmethanesulfonyl fluoride (PMSF) on ice. Lysates were collected by centrifugation at 14,000 g for 5 min at 4 ℃ and the protein concentration was detected using a BCA protein assay kit. Protein samples were separated by sodium dodecyl sulfate polyacrylamide gel electrophoresis (SDS-PAGE) and transferred onto polyvinylidene fluoride (PVDF) membrane. After blocking with 5% non-fat milk in TBS-T for 1 h, the membranes were incubated overnight at 4 °C with primary antibodies against CTGF (Abcam, USA), Smad4, β-actin, DNMT3a, HDAC1, PHB2 (Cell Signaling Technology, Danvers, MA, USA). The goat-anti-rabbit IgG conjugated to horseradish peroxidase (HRP)(1:5000, Cwbiotech, Shanghai, China) were used as the secondary antibody. The signals of protein bands were visualized by ECL method. Anti-β-actin antibody was used as loading control.

**Enzyme-linked immunoabsorbent assay (ELISA)**

HCC cells were stimulated with TGF-β1(10 ng/ml) for 24 h after transfection with miR-34a-5p, miR-34a-5p kd, Smad4, shSmad4 or negative control. The concentrations of IL-11 in culture supernatants were measured with ELISA kits (R & D Systems, Minneapolis, MN, USA) according to the manufacturer’s instructions. The absorbance was determined at 450 nm using a microplate reader.

**Dual luciferase reporter assay**

HEK293T cells were seeded in a 96-well plate at 50% to 60% confluence. After 24 hours, cells were transfected with miR-34a mimics(50nM), miR-34a inhibitor (100nM), or negative control. And cells were cotransfected with 100 μg of the wild-type or mutant 3’-UTR of Smad4. Cells were collected 48 hours after transfection, and the luciferase reporter analysis was conducted in the Luciferase® Reporter Assay System (Promega, Madison, WI, USA) as the manufacturer’s protocol. Luciferase activity was detected using a Microplate Spectrophotometer (Bio-Rad, Hercules, CA, USA).

**Bisulfate-sequencing PCR**

In brief, genomic DNA was obtained from HCC cells with genome isolation kit (Tiangen, China). For bisulfate-sequencing PCR assay, bisulfate treatment and PCR amplification were performed in accordance with the instructions. Primers design, clones construction and selection, and sequencing were performed by Songon Biotech (Shanghai, China).

**RNA pull-down assay**

RNA pull-down assay was performed by using the Pierce™ Magnetic RNA-Protein

Pull-Down Kit (Thermo Fisher Scientific, Waltham, MA,USA). And a nuclease-free environment was maintained during this procedure. Briefly, cell lysates were prepared using standard immunoprecipitation(IP) lysis buffers (Thermo Fisher Scientific, Waltham, MA,USA). The streptavidin magnetic beads were washed and then binded with Lnc34a probe labeled with 3’-biotin. After RNA binding, the cell lysates were incubated with the above beads in Protein-RNA Binding Buffer at 4°C with rotation for the binding of RNA-Binding Proteins(RBP) to RNA. Then the RNA-binding protein complexes were released from the beads through stringent washes and elution. The retrieved samples were collected for Western blotting verification.

**RNA immunoprecipitation (RIP) and chromatin immunoprecipitation (ChIP) assays**

RIP assay was performed by using Protein Immunoprecipitation Kit (Millipore) following the manufacturer’s instructions. Briefly, the cells were lysed by RIP lysis buffer and then maintained with RIP buffer supplemented with magnetic beads conjugated with human DNMT3a, HDAC1, and PHB2 antibody (Cell signal technology, USA) and normal mouse IgG (negative control; Millipore). Then the co-precipitated RNA was further purified and determined by qRT-PCR.

ChIP was performed using a ChIP assay kit (Millipore) according to the manufacturer’s instructions. The histone modifications associated with the miR-34a promoter were analysed by using antibodies against acetylated histones H3 and H4 (Millipore).

**Mice**

Male BALB/c nude mice, 4-6 weeks old, were purchased from Shanghai Slac Laboratory Animal Co. Ltd and maintained in laminar flow cabinets under specific pathogen free (SPF) conditions. All protocols for mouse experiments were approved by the Animal Ethics Committee of Zhongshan Hospital, Fudan University.

**Animal operations**

Cells at 80-90% confluence were harvested and resuspended in ice-cold D-PBS at a concentration of 10^7^ cells per ml. Mice were anesthetized with 1% pentobarbital sodium (45 mg per kg of body weight) before injection. A total of 100 μL suspended cells was injected into the left cardiac ventricle of anesthetized nude mice using a 29-gauge needles within 30 seconds. The entry of right red arterial blood into the syringe indicated a successful injection.

**Bioluminescence assay**

Bioluminescence (BLI) assay was performed using a cryogenically cooled imaging system (CARESTREAM Image Station System, USA). Mice were injected with a fresh stock solution of D-Luciferin, Firefly, potassium salt in DPBS (150 mg/kg body weight, PerkinElmer, Inc. Boston, MA, USA) intra-peritoneally. Then in vivo imaging was performed 10-15 min after injection. And then imaged in prone position. A success of intracardiac injections was identified by the distribution of luminescence signal around the body of the mice 30 min after injection. In vivo BLI assays were serially performed on day 21, 35, and 49 after the injection. Images of the animals were acquired using Living Image software (Xenogen, Corporation, Alameda, CA) by measuring photon flux. The distribution of the photons was indicated by a pseudo color overlay on the gray scale image. And the BLI signal intensity was quantified as the sum of photons within a region of interest (ROI).

**SPECT bone imaging**

The existence of bone metastasis of mice indicated by BLI assay was further examined via SPECT scanning 7 weeks after intracardiac injection. For each mouse, approximately 0.5-1mCi ^99m^Tc-MDP was injected through the tail vein. Bone scanning images were acquired 4-6 h after radiotracer injection using NanoSPECT imaging instrument.

**Micro-computed tomography (Micro-CT) scan**

The bioluminescence-suspected bones were scanned using a Quantum GX microCT Imaging System (PerkinElmer, Inc. Boston, MA, USA). Three-dimensional images were reconstructed and analyzed for the visualization of osteolytic or osteoblastic lesions.

**Pathology analysis**

Bones excised from animals were fixed in 4% paraformaldehyde overnight and subsequently decalcified in EDTA Decalcified Solution (BBI Life sciences) for 2-3 weeks. And the decalcified bones were then sliced using a microtome (Leica, Solms, Germany) with a diameter about 0.4μm. Morphological examination was performed with hematoxylin and eosin (H&E) staining for histological assessment of the bone metastasis progression.

**Tartrate-resistant acid phosphatase (TRAP)**

Tartrate-resistant acid phosphatase (TRAP) staining was performed to identify and quantify osteoclast-like cells using the Acid Phosphatase, Leukocyte (TRAP) Kit (Sigma-Aldrich, Saint Louis, MO) in accordance with the manufacturer's instructions. The number of TRAP-positive multinucleated cells (containing three or more nuclei) was counted using an Olympus microscope.

**References**

[1] Xiang ZL, Zeng ZC, Tang ZY, Fan J, He J, Zeng HY, Zhu XD. Potential prognostic biomarkers for bone metastasis from hepatocellular carcinoma. Oncologist. 2011;16:1028-39.

[2] Zhang L, Xiang ZL, Zeng ZC, Fan J, Tang ZY, Zhao XM. A microRNA-based prediction model for lymph node metastasis in hepatocellular carcinoma. Oncotarget. 2016;7:3587-98.

[3] Hernanda PY, Chen K, Das AM, Sideras K, Wang W, Li J, Cao W, Bots SJ, Kodach LL, de Man RA, et al. Smad4 exerts a tumor-promoting role in hepatocellular carcinoma. Oncogene. 2015;34:5055-68.

[4] He M, Gao L, Zhang S, Tao L, Wang J, Yang J, Zhu M. Prognostic significance of miR-34a and its target proteins of FOXP1, p53, and BCL2 in gastric MALT lymphoma and DLBCL. Gastric Cancer. 2014;17:431-41.

[5] Zhu XD, Zhang JB, Zhuang PY, Zhu HG, Zhang W, Xiong YQ, Wu WZ, Wang L, Tang ZY, Sun HC. High expression of macrophage colony-stimulating factor in peritumoral liver tissue is associated with poor survival after curative resection of hepatocellular carcinoma. J Clin Oncol. 2008;26:2707-16.

[6] Kang Y, He W, Tulley S, Gupta GP, Serganova I, Chen CR, Manova-Todorova K, Blasberg R, Gerald WL, Massague J. Breast cancer bone metastasis mediated by the Smad tumor suppressor pathway. Proc Natl Acad Sci U S A. 2005;102:13909-14.

[7] Wang L, Bu P, Ai Y, Srinivasan T, Chen HJ, Xiang K, Lipkin SM, Shen X. A long non-coding RNA targets microRNA miR-34a to regulate colon cancer stem cell asymmetric division. Elife. 2016;5.
