# Supplementary material for: The molecular mechanism of LncRNA34a-mediated regulation of bone metastasis in hepatocellular carcinoma
Source: Mol Cancer. 2019 Jul 26;18:120. doi: 10.1186/s12943-019-1044-9 (PMC6659280; doi:10.1186/s12943-019-1044-9)
Supplement: Supplementary file 2 — Relationship between lnc34a expression in tissue and clinicopathological characteristics. Table S1. Relationship between lnc34a expression in tissue and clinicopathological characteristics. (DOC 88 kb) [file 12943_2019_1044_MOESM2_ESM.doc]

Table S1 Relationship between lnc34a expression in tissue and clinicopathological characteristics

|  | | Lnc34a expression in tissue | | |
| --- | --- | --- | --- | --- |
| Variable | n | Negative | Positive | P |
| Age |  |  |  |  |
| ≤ 51 | 125 | 62 | 63 | 0.452 |
| >51 | 127 | 69 | 58 |  |
| Gender |  |  |  |  |
| male | 220 | 117 | 103 | 0.318 |
| female | 32 | 14 | 18 |  |
| HBsAg |  |  |  |  |
| negative | 52 | 32 | 20 | 0.122 |
| positive | 200 | 99 | 101 |  |
| HCV-Ab |  |  |  |  |
| negative | 246 | 130 | 116 | 0.181 |
| positive | 6 | 1 | 5 |  |
| AFP |  |  |  |  |
| ≤ 20 | 59 | 35 | 24 | 0.197 |
| > 20 | 193 | 96 | 97 |  |
| ALT |  |  |  |  |
| ≤ 40 | 193 | 99 | 94 | 0.692 |
| > 40 | 59 | 32 | 27 |  |
| γ-GT |  |  |  |  |
| ≤ 50 | 129 | 61 | 68 | 0.126 |
| > 50 | 123 | 70 | 53 |  |
| Liver cirrhosis |  |  |  |  |
| no | 41 | 25 | 16 | 0.208 |
| yes | 211 | 106 | 105 |  |
| Child-Pugh score |  |  |  |  |
| A | 245 | 128 | 117 | 0.915 |
| B | 7 | 3 | 4 |  |
| Tumor differentiation |  |  |  |  |
| I–II | 202 | 111 | 91 | 0.058 |
| III–IV | 50 | 20 | 30 |  |
| Tumor size, cm |  |  |  |  |
| ≤ 5 | 127 | 65 | 62 | 0.797 |
| > 5 | 125 | 66 | 59 |  |
| Tumor number |  |  |  |  |
| single | 203 | 103 | 100 | 0.421 |
| multiple | 49 | 28 | 21 |  |
| Tumor encapsulation |  |  |  |  |
| complete | 130 | 79 | 51 | 0.004 |
| none | 122 | 52 | 70 |  |
| Vascular invasion |  |  |  |  |
| no | 209 | 127 | 82 | <0.001 |
| yes | 43 | 4 | 39 |  |
| BCLC stage |  |  |  |  |
| 0-A | 218 | 128 | 90 | <0.001 |
| B-C | 34 | 3 | 31 |  |

HBsAg hepatitis B surface antigen, HCV-Ab hepatitis C virus antibody, AFP a-fetoprotein, ALT alanine aminotransferase, γ-GT γ-glutamyl transferase, BCLC-stage Barcelona Clinic Liver Cancer-stage.
