# Supplementary material for: The molecular mechanism of LncRNA34a-mediated regulation of bone metastasis in hepatocellular carcinoma
Source: Mol Cancer. 2019 Jul 26;18:120. doi: 10.1186/s12943-019-1044-9 (PMC6659280; doi:10.1186/s12943-019-1044-9)
Supplement: Supplementary file 3 — Multivariate analyses of factors associated with bone metastasis in 252 HCC patients. Table S2. Multivariate analyses of factors associated with bone metastasis in 252 HCC patients. (DOC 30 kb) [file 12943_2019_1044_MOESM3_ESM.doc]

Table S2 Multivariate analyses of factors associated with bone metastasis in 252 HCC patients

| Variable | Bone metastasis | |
| --- | --- | --- |
|  | HR (95% CI) | *P* |
| Tumor encapsulation (complete versus none) | 1.811（0.748-4.386） | 0.188 |
| Vascular invasion (no versus yes) | 3.585（1.562-8.232） | 0.003 |
| BCLC stage ( 0-A versus B-C) | 6.050(2.682-13.651) | <0.001 |
| Lnc34a ( negative versus positive) | 3.306（1.087-10.054） | 0.035 |

HCC hepatocellular carcinoma, BCLC-stage Barcelona Clinic Liver Cancer-stage.
