# Supplementary figures and images for: The molecular mechanism of LncRNA34a-mediated regulation of bone metastasis in hepatocellular carcinoma
Source: Mol Cancer. 2019 Jul 26;18:120. doi: 10.1186/s12943-019-1044-9 (PMC6659280; doi:10.1186/s12943-019-1044-9)

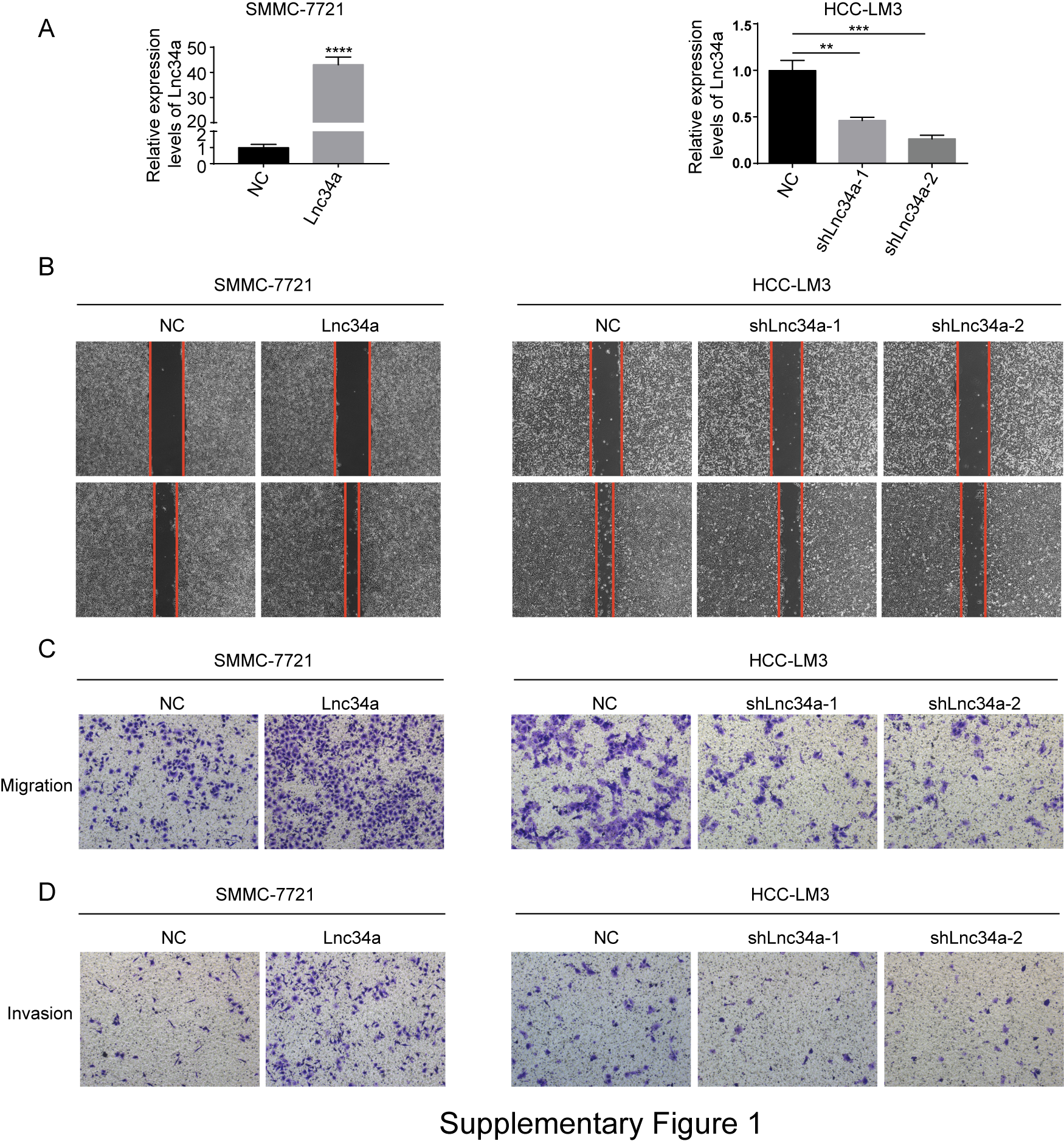

Supplement: Supplementary file 4 — The transfection efficiency of Lnc34a and typtical figures of wound healing assays and transwell assays with or without Matrigel. Figure S1. The validation of Lnc34a or shLnc34a transfections in HCC cell lines (n = 3). The transfection efficiency of Lnc34a or shLnc34a was conducted in SMMC-7721 and HCC-LM3 cells using qRT-PCR (A). The wound-healing assay (B; Magnification: × 40) and transwell assay (Magnification: × 100) without (C) or with Matrigel (D) were performed to analyze the effect of Lnc34a on the migration and invasion of SMMC-7721 and HCC-LM3 cells. NC, negative control. **P < 0.01; ***P < 0.001; ****P < 0.001. (TIF 3.33 MB) [file 12943_2019_1044_MOESM4_ESM.tif]

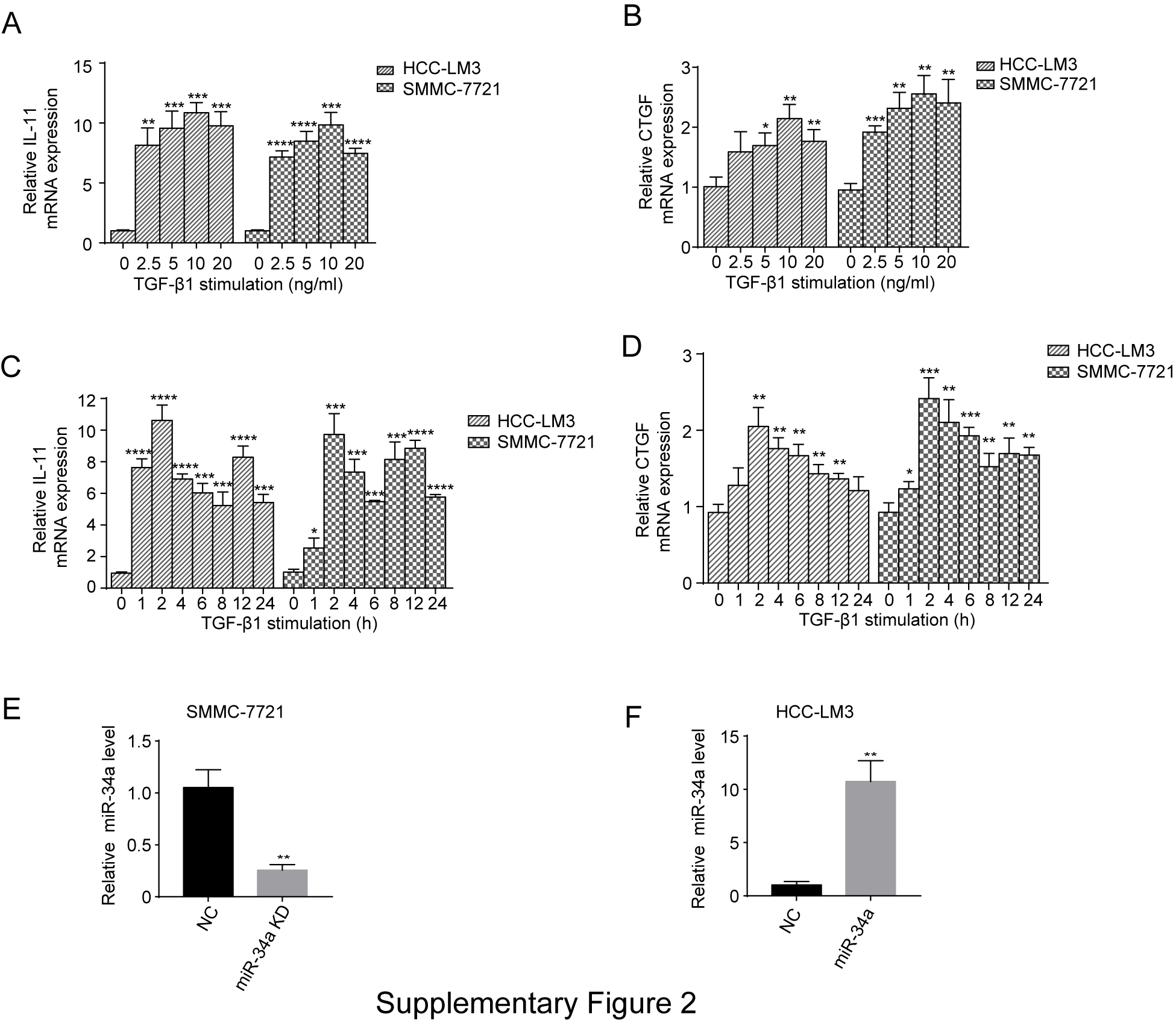

Supplement: Supplementary file 5 — The activation of IL-11 and CTGF by TGF-β in HCC cells. Figure S2. The activation of IL-11 and CTGF by TGF-β in HCC cells (n = 3). (A) SMMC-7721 and HCC-LM3 cells were treated with different amounts of TGF-β1 (approximately 0–20 ng/ml) for 2 h followed by qRT-PCR was used to detect the expression of IL-11 and CTGF; (B) IL-11 and CTGF expression was examined by qRT-PCR following treatment with 10 ng/ml TGF-β1 for the various time periods (0, 1, 2, 4, 6, 8, 12, and 24 h). Modulation of miR-34a expression in SMMC-7721 and HCC-LM3 cells. The different levels of miR-34a expression in transfected SMMC-7721 (E) and HCC-LM3 cells (F) were respectively confirmed by qRT-PCR. NC, negative control. *P < 0.05; **P < 0.01; ***P < 0.001; ****P < 0.001. (TIF 606 KB) [file 12943_2019_1044_MOESM5_ESM.tif]

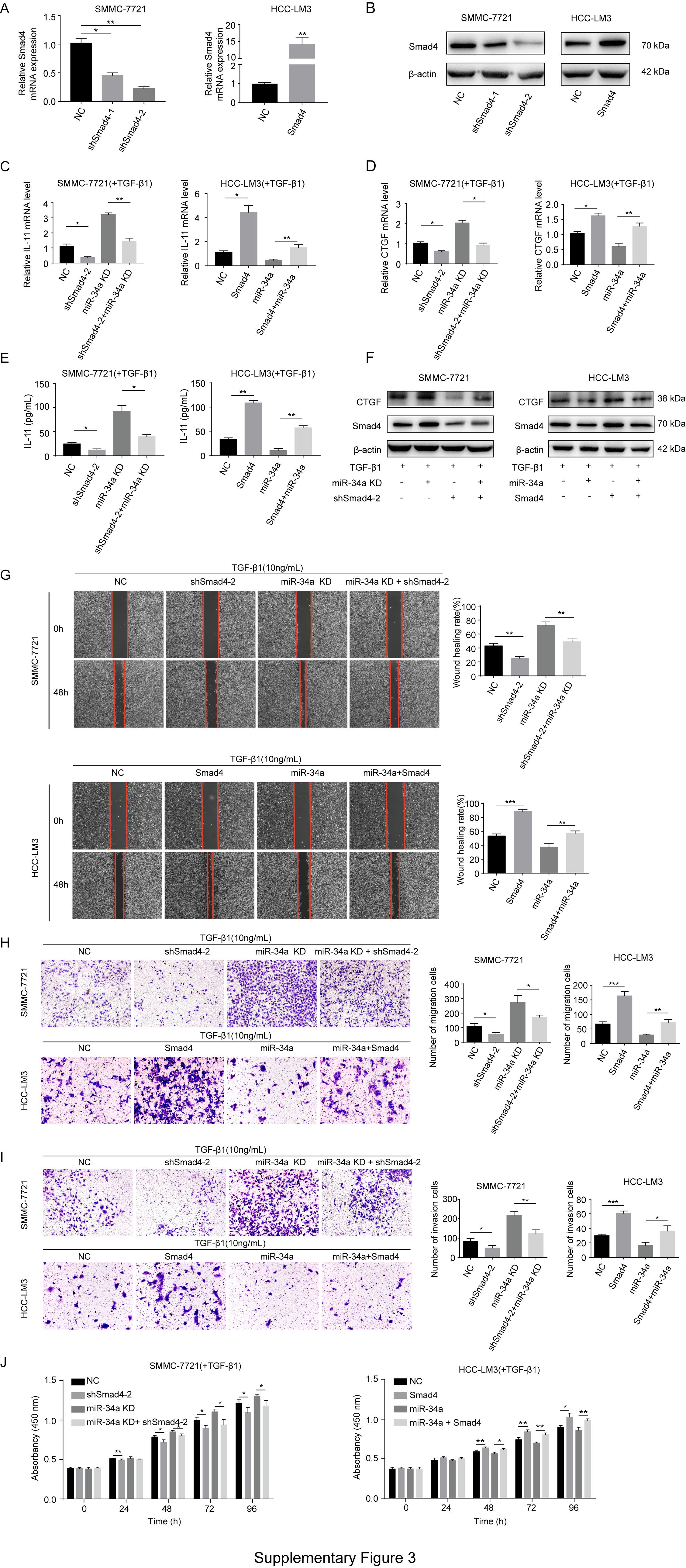

Supplement: Supplementary file 6 — MiR-34a targets Smad4 to inhibit TGF-β1-induced target expression and migration of hepatoma cells. Figure S3. MiR-34a targets Smad4 to inhibit TGF-β1-induced target expression and migration of hepatoma cells (n = 3). Validation of transfection expression in SMMC-7721 and HCC-LM3 cells (A and B). qRT-PCR (C and D), ELISA (E), Western blot (F), wound-healing assay (G; Magnification × 40) and transwell assay (Magnification: × 100) without (H) or with (I) Matrigel and CCK-8 assay (J), were conducted in SMMC-7721 cells transfected with miR-34a kd, shSmad4, miR-34a kd plus shSmad4 or NC, and in HCC-LM3 cells transfected with miR-34a, Smad4, miR-34a plus Smad4 or NC, induced with TGF-β1. NC, negative control. *P < 0.05; **P < 0.01; ***P < 0.001. (TIF 6.18 MB) [file 12943_2019_1044_MOESM6_ESM.tif]
